# Constituents from the Fruiting Bodies of *Trametes cubensis* and *Trametes suaveolens* in Vietnam and Their Anti-Inflammatory Bioactivity

**DOI:** 10.3390/molecules26237311

**Published:** 2021-12-02

**Authors:** Yue-Chiun Li, Nguyen Thi Ngan, Kun-Ching Cheng, Tsong-Long Hwang, Tran Dinh Thang, Nguyen Ngoc Tuan, Mei-Lin Yang, Ping-Chung Kuo, Tian-Shung Wu

**Affiliations:** 1 School of Pharmacy, College of Medicine, National Cheng Kung University, Tainan 701, Taiwan; ycli0126@gmail.com (Y.-C.L.); l3891104@nckualumni.org.tw (M.-L.Y.); 2 Institute of Biotechnology and Food Technology, Industrial University of Ho Chi Minh City, Ho Chi Minh City 700000, Vietnam; nguyenthingan_vsh@iuh.edu.vn (N.T.N.); thangtd@iuh.edu.vn (T.D.T.); nguyenngoctuan@iuh.edu.vn (N.N.T.); 3 Taiwan Sugar Research Institute, Tainan 70176, Taiwan; a64128@taisugar.com.tw; 4 Graduate Institute of Natural Products, College of Medicine, Chang Gung University, Taoyuan 33302, Taiwan; htl@mail.cgu.edu.tw; 5 Research Center for Chinese Herbal Medicine, Research Center for Food and Cosmetic Safety, Graduate Institute of Health Industry Technology, College of Human Ecology, Chang Gung University of Science and Technology, Taoyuan 33302, Taiwan; 6 Department of Anesthesiology, Chang Gung Memorial Hospital, Taoyuan 33302, Taiwan

**Keywords:** *Trametes cubensis*, *Trametes suaveolens*, anti-inflammatory, superoxide anion generation, elastase release

## Abstract

It is reported that various fungi have been used for medicine and edible foods. The tropical *Trametes* genus is popular and well-known in Vietnam for its health effects and bioactivities. In this study, the fruiting bodies of the edible fungi *T. cubensis* and *T. suaveolens* were collected in Vietnam. The preliminary bioactivity screening data indicated that the methanol extracts of the fruiting bodies of *T. cubensis* and *T. suaveolens* displayed significant inhibition of superoxide anion generation and elastase release in human neutrophils. Therefore, the isolation and characterization were performed on these two species by a combination of chromatographic methods and spectrometric analysis. In total, twenty-four compounds were identified, and among these (**1–3**) were characterized by 1D-, 2D-NMR, and HRMS analytical data. In addition, the anti-inflammatory potentials of some purified compounds were examined by the cellular model for the inhibition of superoxide anion generation and elastase release in human neutrophils. Among the isolated compounds, (**5**,**14**), and (**19**) displayed significant anti-inflammatory potential. As the results suggest, the extracts and isolated compounds from *T. cubensis* and *T. suaveolens* are potential candidates for the further development of new anti-inflammatory lead drugs or natural healthy foods.

## 1. Introduction

Once the human body is stimulated by bacteria, viruses, wounds, or other environment factors, the immune system will respond by inflammation to resist the infection and irritation. When organs and tissues are in inflammation, neutrophils are usually the first lymphocytes to reach the infected region [1]. Various cytotoxins, such as superoxide anion and elastase can be secreted by neutrophils in response to activation of the immune system [2]. Neutrophil overexpression has been demonstrated to be related to many human diseases in recent years [3,4,5,6,7]. Coussens et al. showed the relationship between inflammation and cancer, and they also indicated that the formation of cancer cells is attributed to inflammation [1]. Therefore, compounds with anti-inflammatory bioactivity are potential candidates for further discoveries related to treatment of cancers. In neutrophils, superoxide anion generation and elastase release are activated by N-formyl-methionyl-leucyl-phenylalanine (fMLF) and cytochalasin B (CB) and anti-inflammatory bioactivity can be evaluated with the addition of analytes. Therefore, the cellular neutrophil model is an effective system for the screening of anti-inflammatory drugs. Chinese herbal medicine has been used for thousands of years, and it is believed to display relatively lower side effects on the human body. Thus, the application of Chinese herbal medicine to the development of anti-inflammatory lead drugs for the treatment of immune diseases and cancer has become popular in recent years.

Many fungi have been reported for their healthy values, such as *Ganoderma lucidum, Trametes versicolor, Sanghuangporus sanghuang, Wolfiporia extensa*, and *Ophiocordyceps sinensis*. Medicinal fungi such as *Ganoderma lucidum* and *Taiwanofungus camphoratus* are rich in polysaccharides and triterpenoids. It is believed in traditional medicine that these medicinal fungi possess detoxification, blood circulation, immunity enhancement, anti-inflammation, anti-cancer, and longevity effects [8,9,10,11,12,13,14]. Basidiomycota is a class of higher fungi, containing more than 20,000 species, including mushrooms and other major edible fungi. The most well-known Basidiomycota fungi is Polyporales, and some species such as *Trametes versicolor* and *Fomes fomentarius*, have already been used in Chinese medicine. In addition, *Lentinus*, *Fomitopsis*, and *Ganoderma* are also extensively used in food and medicine [15,16,17,18,19,20,21,22,23,24,25].

**Figure 1:**
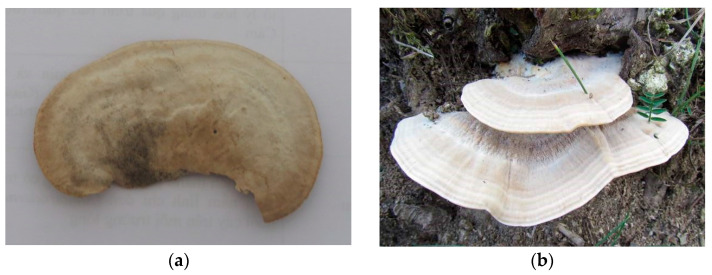
Fruiting bodies of (**a**) *T. cubensis* and (**b**) *T. suaveolens*.

*Trametes cubensis* (Mont.) Sacc. and *T. suaveolens* (Figure 1) are commonly used edible fungi in Vietnam and belong to the *Trametes* genus. They mainly grow on living or dead trees. The fruiting bodies of these fungi display various shapes, white color, and are types of saprophytic fungi. The research reports of the *Trametes* genus mostly focus on *T. versicolor* but other species are relatively few. Walder et al. reported the water extracts of *T. cubensis* exhibited strong anti-HIV-1 activity [26]. In addition, *T. suaveolens* demonstrated antioxidant [27], anti-complementary [28], and anti-HIV-1 [29] bioactivities. In preliminary examination, the methanol extracts of fruiting bodies of *T. cubensis* and *T. suaveolens* were subjected to evaluation for their anti-inflammatory bioactivities. The results that *T. cubensis* promoted superoxide anion generation and elastase release, indicated the immunostimulatory effect (Table 1). Therefore, in the present study the bioactive constituents of fruiting bodies of *T. cubensis* and *T. suaveolens* were investigated and the purified compounds were evaluated for their anti-inflammatory bioactivity in a cellular neutrophil model. Hopefully, the extracts and isolated compounds from the fruiting bodies of *T. cubensis* and *T. suaveolens* can be developed as lead compounds in anti-inflammatory drugs as well as natural ingredients of healthy foods.

## 2. Results and Discussion

**Figure 2:**
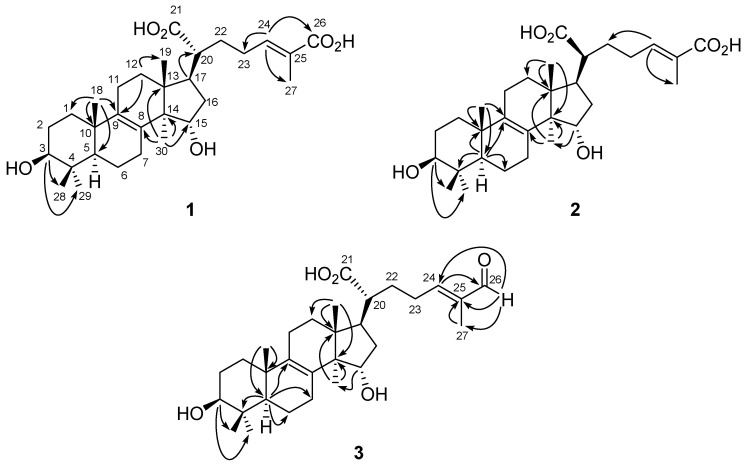
Structures and key HMBC correlations (→) of compounds **1**–**3**.

The fruiting bodies of *T. cubensis* and *T. suaveolens* were extracted with methanol, and then partitioned with dichloromethane and water to give aliphatic and water soluble layers. The dichloromethane layer was subjected to purification by the combination of conventional chromatographic techniques. In *T. cubensis*, two new triterpenoids (**1**–**2**) (Figure 2) were characterized and their chemical structures were constructed with the assistance of the NMR spectral elucidation and MS spectrometric analysis. Moreover, fourteen known compounds, including eight triterpenoids, hexagonin F (**3**) [30], sinensoic acid (**4**) [31], 24(*E*)-3*β*-hydroxylanosta-8,24-dien-26-al-21-oic acid (**5**) [32], trametenolic acid (**6**) [33], eburicoic acid (**7**) [34], 24-methylenelanost-8-en-3-ol (**8**) [35], eburicodiol (**9**) [36], and gloeophyllin B (**10**) [37]; four steroids, ergosterol peroxide (**11**) [38], ergosterol (**12**) [39], (22*E*)-ergosta-7,22-dien-3*β*-ol (**13**) [40], and (22*E*,24*R*)-5-hydroxy-5*α*-ergosta-7,22-diene-3,6-dione (**14**) [41]; and two isocoumarins, oospoglycol (**15**) [42] and oospolactone (**16**) [43], were identified. In addition, eleven known constituents, including one triterpenoid, lupeol (**17**) [44]; seven steroids, ergosterol peroxide (**11**), (22*E*)-ergosta-7,22-dien-3*β*-ol (**13**), (22*E*,24*R*)-5-hydroxy-5*α*-ergosta-7,22-diene-3,6-dione (**14**), 9,11-dehydroergosterol peroxide (**18**) [45], 5,8-epidioxy-(5*α*,8*α*,22*E*)-ergosta-6,22-dien-3-one (**19**) [46], ergosta-7,22-diene-3-one (**20**) [40], and stigmast-4-en-3-one (**21**) [47]; and three benzenoids, syringic acid (**22**) [48], isovanillic acid (**23**) [49], and tyrosol (**24**) [50], were characterized from the fruiting bodies of *T. suaveolens*. The known compounds were identified by comparison of their physical and spectroscopic data with those previously published.

### 2.1. Structural Elucidation of Compounds ***1*–*3***

**Figure 3:**
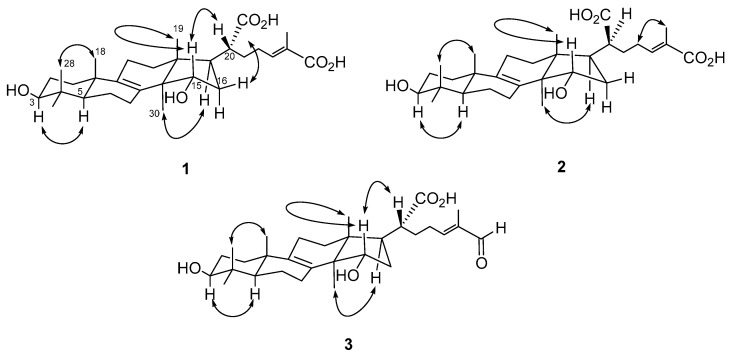
Key NOESY correlations (↔) of compounds **1**–**3**.

Compound **1** was isolated as optically active white powder, and the molecular formula was assigned as C_30_H_46_O_6_ by HR-ESI-MS analysis ([M − H]^−^, *m*/*z* 501.3216, calculated for C_30_H_45_O_6_, 501.3216, Figure S1). The IR spectrum indicated the presence of an hydroxyl (3406 cm^−1^) and a conjugated carbonyl group (1674 cm^−1^). There were six methyl singlets in ^1^H-NMR spectrum (Figure S2) at 0.80 (3H, CH_3_-28), 0.85 (3H, CH_3_-19), 0.93 (3H, CH_3_-30), 0.98 (3H, CH_3_-29), 0.99 (3H, CH_3_-18), and 1.79 (3H, CH_3_-27), which revealed the structure of **1** to be a lanostanoid triterpene. In addition, the characteristic proton signals at δ 3.15 (1H, t, *J* = 7.2 Hz, H-3), 4.16 (1H, dd, *J* = 9.6, 6.0 Hz, H-12), and 6.62 (1H, td, *J* = 7.2, 1.2 Hz, H-24) indicated the presence of hydroxy substitutions, and carbon–carbon double bonds (Table 2). According to its ^13^C-NMR, DEPT, and HSQC spectra (Figures S3 and S4), there were two oxygenated methines at δ 74.3 (C-15) and 79.7 (C-3), two quaternary carbons which are tetrasubstituted double bond at δ 135.6 (C-9) and 136.2 (C-8), one set of trisubstituted double bond at δ 134.7 (C-25) and 137.2 (C-24), and one carboxylic acid group at δ 178.1 (C-26). The *^2^J-* and *^3^J-* HMBC correlations (Figure S5) from H-3 to CH_3_-28 and CH_3_-29; from H-12 to C-9 and CH_3_-19; from H-17 to C-20; from CH_3_-18 to C-1, C-5, C-9, and C-10; from H-24 to C-23, C-26, C-27; and from CH_3_-30 to C-8, C-13, C-14, and C-15, constructed the planar structure of **1** (Figure 2). The configuration of OH-3 was determined as β according to the coupling constant of H-3 (7.2 Hz). Comparing the NOE correlations (Figure S6) of H-3/H-5, CH_3_-18/CH_3_-28, H-17/CH_3_-30, H-20/H-15, CH_3_-19, and H-16β, with those previously reported [51,52,53] suggested the configuration of **1** as shown (Figure 3). Moreover, the downfield chemical shift of H-24 [40,54] and no NOE correlation could be observed between H-24 and CH_3_-27 in the NOESY spectrum which indicated the configuration of C-24/C-25 as *E* form. Conclusively, the complete structure of **1** was established as shown (Figure 2) and named trivially as trametin A. 

Trametin B (**2**) was isolated as a white solid and its molecular formula was also proposed as C_30_H_46_O_6_ based on a deprotonated molecular ion peak at *m*/*z* 501 in the ESI-MS analysis (Figure S7), which was the same as that of **1** and suggested **2** was an isomer of **1**. The absorptions in the IR spectrum (3434 and 1629 cm^−1^) implied the hydroxy and conjugated carbonyl moieties, respectively. The ^1^H-NMR spectrum of **2** (Figure S8) revealed almost identical spectral characteristics of a lanostanoid triterpene to those of **1** (Table 2). The significant HMBC correlations (Figure S11) from H-3 to CH_3_-28 and CH_3_-29; from H-5 to C-4, C-6, C-9, and C-10; from H-15 to CH_3_-30; from CH_3_-18 to C-5, C-9, and C-10; from CH_3_-19 to C-12, C-13, and C-14; from H-24 to C-22 and C-27; and from CH_3_-30 to C-8, C-13, and C-14, established the planar structure of **2** as the same as that of **1** (Figure 2). The large coupling constants of H-3 (*J* = 8.0, 7.2 Hz) indicated the orientation was *β* form. The NOE effects (Figure S12) of H-3/H-5, H-14/H-17, H-15/CH_3_-19, CH_3_-18/CH_3_-28, and H-23/CH_3_-27 supported the configuration of **2** as shown (Figure 3), which was 20*S* rather than the common 20*R*. In addition, the NOE correlation between H-23 and CH_3_-27 also supported the *E* configuration at C-24/C-25. Conclusively, the structure of **2** was established as a stereoisomer of **1** based on the spectral data and its structure was provided (Figure 2).

Compound **3** was obtained as a white powder and its molecular formula was assigned as C_30_H_46_O_5_ on the basis of HR-ESI-MS analytical data (*m*/*z* 485.3263 [M − H]^−^) (Figure S13). The IR absorption peaks at 3368 and 1658 cm^−1^ indicated the hydroxy and carbonyl functionalities. The ^1^H-NMR data of **3** (Figure S14) showed much similarity with those of **1**, except for the loss of a carboxyl group and the presence of an aldehyde moiety in **3** (δ_H_ 9.36 and δ_C_ 197.3) (Table 2). All the ^1^H- and ^13^C-NMR data were coincided well with those reported for hexagonin F [30]. However, the clear HMBC spectral analytical data revealed the different substituted pattern as compared with the previous paper. The ^2^*J*- and ^3^*J*-correlation peaks (Figure S17) from H-3 to CH_3_-28 and CH_3_-29; from H-5 to C-4, C-6, C-7, and C-9; from H-15 to CH_3_-30; from CH_3_-18 to C-5 and C-10; from CH_3_-19 to C-12, C-13, and C-14; from H-26 to C-24, C-25, and CH_3_-27; from H-24 to C-26; from CH_3_-27 to C-25; from CH_3_-30 to C-13 and C-14, were observed in its HMBC spectrum (Figure 2), which suggested it was 3,11-dihydroxylanosta-8,24-dien-26-al-21-oic acid. In the NOESY spectrum (Figure S18), the correlations of H-3/H-5, H-15/CH_3_-19 and H-20, H-17/CH_3_-30, and CH_3_-18/CH_3_-28, established its configuration as the same as that of **1**. According to the present spectral data, the chemical structure of **3** should be revised as shown (Figure 2).

### 2.2. Anti-Inflammatory Activity

**Figure 4:**
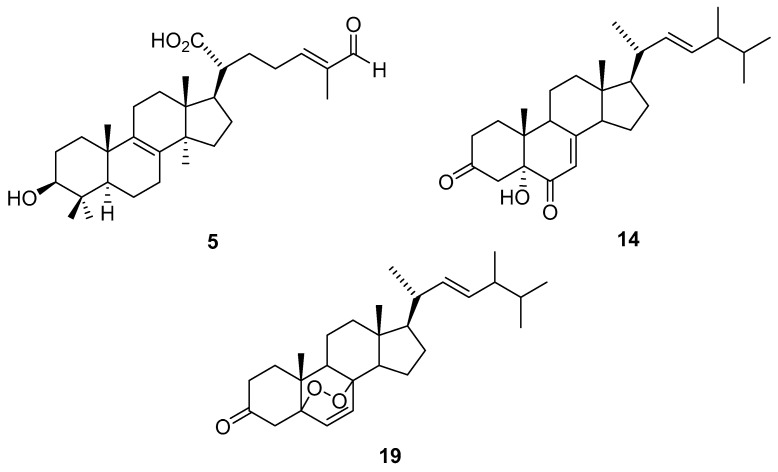
Chemical structures of **5**, **14**, and **19**.

The isolated compounds which were found to be more abundant in the present study were subjected to evaluation of their inhibitory activity against superoxide anion generation and elastase release by human neutrophils in response to fMLF/CB (Table 3) [7]. Significant inhibition of superoxide anion generation was demonstrated for **5**, **14**, and **19** (Figure 4) with IC_50_ values ranging from 2.3 ± 0.2 to 4.1 ± 0.4 μM, as compared with the positive control LY294002 (IC_50_ 1.1 ± 0.3 μM). In addition, these compounds **5**, **14**, and **19** also exhibited the potential inhibition of elastase release with IC_50_ values ranged from 4.3 ± 0.2 to 5.2 ± 0.4 μM, as compared to the positive control LY294002 (IC_50_ 3.2 ± 1.0 μM) (Table 3). The anti-inflammatory data revealed multiple anti-inflammatory bioactivities in active components which were purified from the fruiting bodies of *T. cubensis* and *T. suaveolens*. In previous reports, **5** showed growth inhibition on several tumor cell lines (IC_50_ 10–25 μg/mL) [32]. Compound **14** was reported to attenuate the growth and triggered an apoptotic process in prostate cancer cells [55]. Inhibition of various tumor cell lines by compound **19** was also determined [46]. Based on our anti-inflammatory examination results and reported bioactivities in the literature, it can be concluded that the fruiting bodies of *T. cubensis* and *T. suaveolens* could be promising as anti-inflammatory lead drugs or natural, healthy ingredients.

### 2.3. In Silico Study of the Potential Compounds

**Figure 5:**
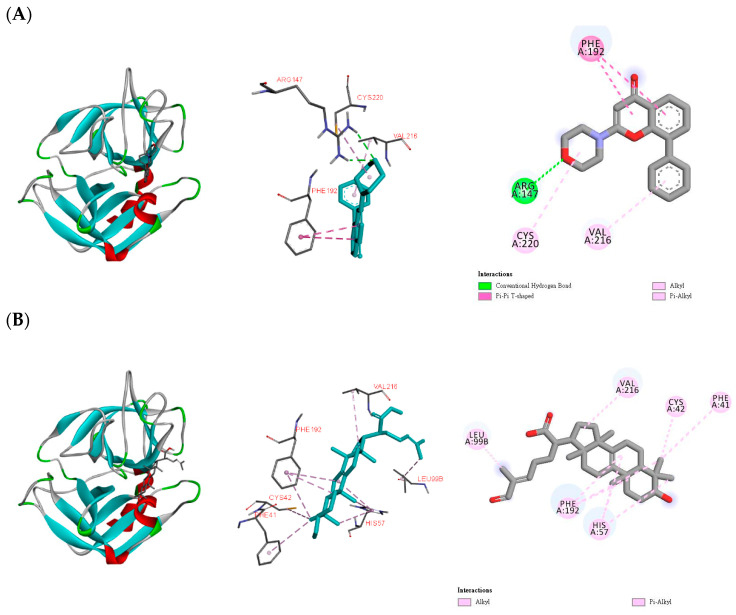

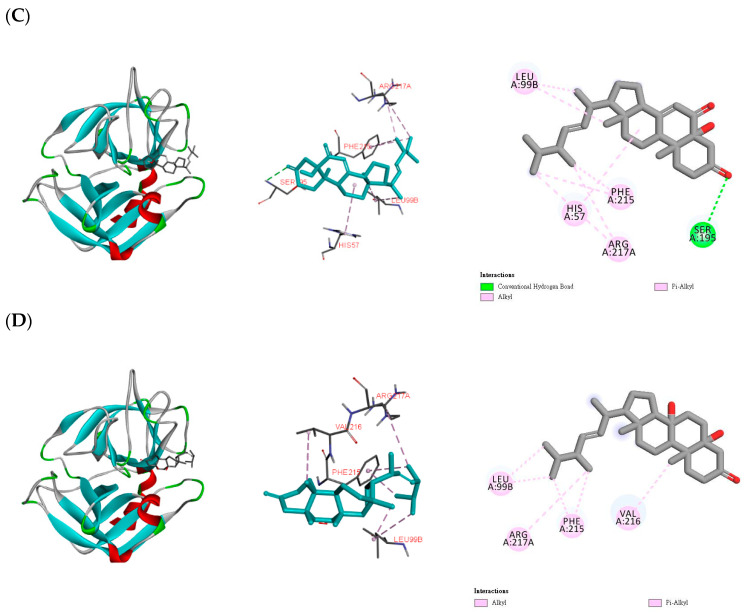
In silico modeling of (**A**) LY294002, (**B**) **5**, (**C**) **14**, and (**D**) **19** docking into the human neutrophil elastase.

According to the bioactivity examination results, compounds **5**, **14**, and **19** exhibited significant inhibition of elastase release. Thus, the three compounds were subjected to computing to evaluate their binding affinity with human neutrophil elastase, which was a trypsin-like serine protease, and played an important role in inflammation [56]. The simulation of elastase and small molecules has been reported [57,58]. The best poses were decided by the lowest binding energy of each ligand. LY294002 was used as a positive control. After simulation, binding energy of LY294002 was determined as −6.0 kcal/mol (Table 4). LY294002 was linked to Arg147 by a hydrogen bond, and to Cys220, Phe192, and Val216 by different effects (Figure 5A). Compounds **5**, **14**, and **19** showed even lower binding energy (Table 4), and this meant that these three compounds could connect to elastase easier than LY294002. Compound **5** and elastase formed a stable complex through alkyl and π-alkyl interactions with Cys42, His57, Leu99B, Phe41, Phe192, and Val216 (Figure 5B). For **14,** the hydrogen bond with Ser195, and other interactions with Arg217A, His57, Leu99B, and Phe215 established the affinity with elastase (Figure 5C). Compound **19** was bound with Arg217A, Lue99B, Phe215, and Val216 via alkyl and π-alkyl interactions (Figure 5D). All these in silico computing results coincided well with those afforded from the biological activity experiments.

## 3. Materials and Methods

### 3.1. General Experimental Procedures

A WRX-4 melting-point apparatus was utilized to record the melting points without correction. The Jasco P-2000 digital polarimeter was used to obtain the optical rotations. The ultraviolet (UV) and infrared (IR) spectra were examined by an Hitachi U-2001 UV/Vis spectrometer and a PerkinElmer FT-IR Spectrum RX1 spectrophotometer, respectively. ^1^H-, ^13^C-, and 2D nuclear magnetic resonance (NMR) spectra were recorded on the Bruker AV-400 NMR spectrometer. Chemical shifts are expressed in *δ* values (ppm) using tetramethylsilane as an internal standard. High-resolution electrospray ionization mass spectrometry (HR-ESI-MS) were examined on a JEOL JMS-700 spectrometer that the experimental data were afforded in the negative-ion mode.

### 3.2. Fungi Material

The fruiting bodies of *T. cubensis* and *T. suaveolens* were collected and identified by Ngo Anh (Department of Biology, Hue University) at the Pumat National Park of Nghean Province, Vietnam, in August 2018. The voucher specimen (Vinh 2018A001 and 2018A002) was deposited at the School of Chemistry, Biology and Enviroment, Vinh University, Vinh City, Vietnam.

### 3.3. Extraction and Isolation

The fruiting bodies of *T. cubensis* (dried weight 1.0 kg) were powdered and extracted with methanol under reflux, the combined extracts then concentrated in vacuo to obtain a brownish syrup (120 g). The methanol extract was partitioned between dichloromethane and water to yield a dichloromethane layer (40 g) and a water layer (80 g).

The dichloromethane layer was resolved on a silica gel column and eluted with a step gradient mixture of chloroform and methanol (50:1 to 1:1) to afford seven fractions (DQE 1–7). The first fraction (DQE 1) was purified by silica gel column chromatography with a gradient mixture of chloroform and ethyl acetate (300:1 to 1:1) to afford six subfractions (DQE 1.1–1.6). The first subfraction (DQE 1.1) was recrystallized and give **16** (2 mg). The fourth subfraction (DQE 1.4) was further column-chromatographed on silica gel with a mixture of chloroform and ethyl acetate (step gradient from 300:1 to 1:1) to obtain seventeen minor fractions (DQE 1.4.1–1.4.17). The fifth minor fraction (DQE 1.4.5) was purified by repeated column chromatography over silica gel eluted with a step gradient mixture of benzene and ethyl acetate (300:1 to 1:1) to result in **8** (3 mg). The minor fraction (DQE 1.4.7) was purified by preparative thin layer chromatography (pTLC) eluted with a solvent mixture of chloroform and acetone (300:1) to produce **11** (6 mg). Compound **13** (2 mg) was yielded from 1.4.9 by pTLC eluted with a solvent mixture of chloroform and acetone (300:1). The eleventh minor fraction (DQE 1.4.11) was purified by silica gel column chromatography with a mixture of hexanes and ethyl acetate (20:1) and further recrystallization of the resulting fractions afforded **9** (2 mg) and **14** (3 mg). The second fraction was isolated by silica gel column chromatography with a gradient mixture of chloroform and acetone (300:1 to 1:1) to produce thirteen subfractions (DQE 2.1–2.13). The subfraction (DQE 2.4) was resolved on a silica gel column and eluted with a step gradient mixture of chloroform and methanol (100:1 to 1:1) to afford nine minor fractions (DQE 2.4.1–2.4.9). The sixth minor fraction (DQE 2.4.6) was purified by silica gel column chromatography with a mixture of chloroform and ethyl acetate (30:1) and further recrystallization of the resulting fractions gave a mixture of **6** (4 mg) and **7** (5 mg). The nineth minor fraction (DQE 2.4.9) was applied to pTLC with a solvent mixture of chloroform and acetone (10:1) to give **15** (3 mg). The fifth subfraction (DQE 2.5) was purified by silica gel column chromatography eluted with a gradient solvent mixture of chloroform and methanol (50:1 to 1:1) to yield seven minor fractions (DQE 2.5.1–2.5.7). The first minor fraction (DQE 2.5.1) was isolated by pTLC eluted with a solvent mixture of chloroform and acetone (30:1) to obtain **10** (3 mg). The fifth minor fraction (DQE 2.5.5) was resolved on a silica gel column and eluted with a step gradient mixture of chloroform and acetone (50:1 to 1:1) to afford fourteen minor subfractions (DQE 2.5.5.1–2.5.5.14). Compound **5** (3 mg) was obtained from 2.5.5.8 by repeated silica gel column chromatography with chloroform and methanol eluent (20:1 to 1:1). DQE 2.5.5.9 was isolated by reversed-phase HPLC with a Gemini 5u C18 column (250 × 4.6 mm, 5μm) eluted with a MeOH–0.5% acetic acid_(aq)_ mixture (62:38, 0.8 mL/min) to yield **3** (15.0 mg). The third fraction (DQE 3) was subjected to crystallization with chloroform and methanol to produce **4** (3 mg). Fraction 4 was isolated by silica gel column chromatography eluted with a step gradient mixture of chloroform and methanol (50:1 to 1:1) to afford seven subfractions (DQE 4.1–4.7). A repeated silica gel column chromatography was performed from 4.1 eluted with a step gradient of chloroform and methanol (50:1 to 1:1) and further recrystallization to give **1** (30.0 mg). The third subfraction (DQE 4.3) was purified by silica gel column chromatography eluted with a step gradient mixture of chloroform and acetone (10:1 to 1:1) to yield nine minor fractions (DQE 4.3.1–4.3.9). Compound **2** (3.0 mg) was obtained from 4.3.9 separated by pTLC eluted with a solvent mixture of chloroform and acetone (3:1). Fraction 7 was recrystallized to obtain **12** (10 mg).

The powdered fruiting bodies of *T. suaveolens* (dried weight 3.0 kg) were extracted by methanol and fractionated with dichloromethane and water to obtain a dichloromethane layer (132 g), a water layer (172 g), and an insoluble layer (36 g).

The dichloromethane layer was subjected to a silica gel column separated by a gradient solvent mixture of chloroform and methanol (300:1 to 1:1) to obtain eight fractions (TED 1–8). The third fraction (TED 3) was further isolated by silica gel column eluted with benzene and a step gradient of acetone (50:1 to 1:1) to afford ten subfractions (TED 3.1–3.10). Compounds **17** (4 mg), **19** (2 mg), and **20** (3 mg) were yielded from 3.7 by repeated silica gel column chromatography with chloroform and methanol eluent (100:1 to 1:1). Nine subfractions (TED 6.1–6.9) were obtained from the sixth fraction by a silica gel column separated with a gradient solvent mixture of chloroform and acetone (300:1 to 1:1). The third subfraction (TED 6.3) was purified by pTLC eluted with a solvent mixture of benzene and ethyl acetate (3:1) to afford **11** (4 mg) and **18** (3 mg). The fourth subfraction (TED 6.4) was resolved on pTLC eluted with a solvent mixture of hexanes and acetone (1:1) to yield **13** (2 mg) and **14** (3 mg). A repeated column chromatography eluted with chloroform and ethyl acetate (step gradient from 50:1 to 1:1) was performed on the eighth subfraction (TED 6.8) to produce **21** (2 mg), **22** (8 mg), **23** (10 mg), and **24** (4 mg).

### 3.4. Spectral and Physical Data of ***1***–***3***

#### 3.4.1. Trametin A (**1**)

White powder; mp: 171.6–173.8 °C; [α]^25^_D_ + 56.6 (*c* 0.3, MeOH); IR (neat) *ν*_max_ 3406, 2938, 1674, 1384, 1259, 1038 cm^−1^; ^1^H and ^13^C NMR, see Table 2; ESI-MS (*rel. int.*): *m*/*z* 501 ([M − H]^−^, 100); HRESIMS *m*/*z* 501.3216 ([M − H]^−^ calculated for C_30_H_45_O_6_, 501.3216).

#### 3.4.2. Trametin B (**2**)

White solid; mp: 166.3–168.5 °C; [α]^25^_D_ − 35.7 (*c* 0.1, MeOH); IR (neat) *ν*_max_ 3434, 2923, 1629, 1560, 1395, 1096 cm^−1^; ^1^H and ^13^C NMR, see Table 2; ESI-MS (*rel. int.*): *m*/*z* 501 ([M − H]^−^, 30), 325 (100).

#### 3.4.3. Hexagonin F (**3**)

White powder; mp: 163.9–165.3 °C; [α]^25^_D_ + 13.2 (*c* 0.2, MeOH); IR (neat) *ν*_max_ 3368, 2930, 1658, 1570, 1394, 1036 cm^−1^; ^1^H and ^13^C NMR, see Table 2; ESI-MS (*rel. int.*): *m*/*z* 485 ([M − H]^−^, 100); HRESIMS *m*/*z* 485.3263 ([M − H]^−^ calculated for C_30_H_45_O_5_, 485.3267).

### 3.5. Anti-Inflammatory Bioactivity Examination

The present study of human neutrophils was approved by the Chang Gung Memorial Hospital Institutional Review Board (No. 1612200032, Taoyuan, Taiwan) and was conducted according to the Declaration of Helsinki (2013). The examination for the superoxide anion and elastase release inhibition was based on the superoxide dismutase (SOD)-inhibitable reduction of ferricytochrome c and degranulation of azurophilic granules as reported [59]. Detailed procedures are provided in the Supplementary Materials S1.

### 3.6. Molecular Docking Study

The AutoDock Vina program was performed for in silico calculation [60]. The crystal structure of human neutrophil elastase has been clarified [61], the PDB file was obtained from the Protein Databank (PDB ID: 1H1B). The B chain of protein was deleted, as well as the water molecules (without hydrogen bonds). The three-dimensional structures of ligands were established by Chem3D program. The AutoDockTools (ADT ver. 1.5.6) was constructed for hydrogen supplement, Gasteiger charge measurement, and flexible torsions. The size of the grid (15.0 Å × 15.0 Å × 15.0 Å) and grid center (18.6, 11.8, 22.8) were designed according PDB file of 1H1B. Docking scores was shown in kcal/mol and represent binding affinity energy. Only the top-scoring pose was considered the best interaction. The interactions between protein and ligands were visualized by Biovia Discovery Studio client 2020 (Dassault Systèmes BIOVIA, Discovery Studio Modeling Environment, Release 2017, San Diego: Dassault Systèmes, 2016).

## 4. Conclusions

Sixteen ingredients, including two new compounds and a revised structure constituent, were isolated from the methanol extracts of fruiting bodies of *T. cubensis*. In addition, eleven constituents were characterized from the fruiting bodies of *T. suaveolens*. Their chemical structures were characterized via spectroscopic and spectrometric analyses. In total, eleven purified compounds were evaluated for their anti-inflammatory activity by inhibition of superoxide anion generation and elastase release on a neutrophil model. The examined data demonstrated that **5**, **14**, and **19** display significant anti-inflammatory bioactivities. Therefore, the fruiting bodies of *T. cubensis* and *T. suaveolens* have the potential to be developed as new anti-inflammatory lead drugs or food products.

## Figures and Tables

**Table 1:** Preliminary bioactivity screening of fruiting bodies of *T. cubensis* and *T. suaveolens* on superoxide anion generation and elastase release by human neutrophils in response to fMLF/CB.

Samples	Superoxide Anion Generation		Elastase Release	
	Inhibition (%) ^a^	Promotion (%) ^b^	Inhibition (%)	Promotion (%)
*T. cubensis*	– ^c^	50.9 ± 5.1 ***	–	98.5 ± 7.7
*T. suaveolens*	15.2 ± 3.8 *	–	1.9 ± 4.7	–
	**IC_50_ (μg/mL) ^d^**		**IC_50_ (μg/mL)**	
LY294002 ^e^	0.4 ± 0.02 ***	–	1.5 ± 0.3 ***	–

^a^ Inhibitory percentage at 10 μg/mL sample concentration. Results are presented as mean ± S.E.M. (n = 3 or 4). * *p* < 0.05, *** *p* < 0.001 compared with the control value. ^b^ Promotion of sample on superoxide anion generation and elastase release as compared to fMLF/CB (100%). ^c^ Not determined. ^d^ 50% Inhibitory concentration (IC_50_). ^e^ A phosphatidylinositol-3-kinase inhibitor was used as a positive control for superoxide anion generation and elastase release.

**Table 2:** ^1^H and ^13^C NMR spectroscopic data of compounds **1**–**3**.

Position	1		2		3	
	*δ*_H_	*δ*c	*δ*_H_	*δ*c	*δ*_H_	*δ*c
1	1.80 (1H, m) 1.95 (1H, m)	38.8	1.75 (1H, m) 1.90 (1H, m)	39.2	1.76 (1H, m) 1.91 (1H, m)	38.9
2	1.65 (2H, m)	32.4	1.52 (2H, m)	33.1	1.64 (2H, m)	32.4
3	3.16 (1H, dd, *J* = 8.0, 7.2 Hz)	79.6	3.15 (1H, dd, *J* = 8.8, 7.2 Hz)	79.7	3.16 (1H, dd, *J* = 8.8, 7.6 Hz)	79.6
4	–	39.9	–	39.9	–	39.9
5	1.05 (1H, m)	51.8	1.03 (1H, m)	51.9	1.03 (1H, m)	51.9
6	2.20 (2H, m)	28.5	2.18 (2H, m)	28.5	1.62 (1H, m)	28.5
7	1.65 (2H, m)	28.2	1.62 (2H, m)	28.3	2.19 (1H, m) 2.37 (1H, m)	28.2
8	–	135.4	–	135.6	–	135.4
9	–	136.2	–	136.2	–	136.2
10	–	38.3	–	38.3	–	38.3
11	1.60 (1H, m) 1.75 (1H, m)	19.4	1.54 (1H, m) 1.72 (1H, m)	19.5	1.55 (1H, m) 1.74 (1H, m)	19.4
12	1.29 (2H, m)	30.6	1.31 (2H, m)	30.8	1.30 (2H, m)	30.5
13	–	52.6	–	52.6	–	52.6
14	–	46.0	–	46.1	–	46.1
15	4.19 (1H, dd, *J* = 9.2, 5.6 Hz)	73.7	4.16 (1H, dd, *J* = 9.6, 6.0 Hz)	74.3	4.18 (1H, dd, *J* = 9.6, 5.6 Hz)	73.9
16	2.05 (2H, m)	21.7	1.99 (2H, m)	21.8	1.99 (2H, m)	21.8
17	2.20 (1H, m)	47.1	2.18 (1H, m)	47.0	2.19 (1H, m)	47.1
18	1.00 (3H, s)	19.6	0.99 (3H, s)	19.6	1.00 (3H, s)	19.6
19	0.83 (3H, s)	16.8	0.85 (3H, s)	16.2	0.84 (3H, s)	16.2
20	2.20 (1H, m)	49.0	2.18 (1H, m)	47.2	2.19 (1H, m)	51.1
21	–	180.2	–	180.4	–	178.6
22	1.25 (1H, m) 1.75 (1H, m)	37.0	1.20 (1H, m) 1.74 (1H, m)	37.1	1.21 (1H, m) 1.73 (1H, m)	37.0
23	2.20 (2H, m)	27.5	2.18 (2H, m)	28.3	2.37 (2H, m)	28.2
24	6.74 (1H, t, *J* = 6.8 Hz)	142.5	6.45 (1H, td, *J* = 7.2, 1.2 Hz)	137.2	6.63 (1H, t, *J* = 6.8 Hz)	156.6
25	–	129.8	–	134.7	–	140.6
26	–	171.8	–	178.1	9.36 (1H, s)	197.3
27	1.79 (3H, s)	12.5	1.79 (3H, s)	13.9	1.71 (3H, s)	9.1
28	0.80 (3H, s)	16.2	0.80 (3H, s)	17.0	0.80 (3H, s)	16.9
29	0.99 (3H, s)	28.6	0.98 (3H, s)	28.6	0.98 (3H, s)	28.6
30	0.93 (3H, s)	17.8	0.93 (3H, s)	17.9	0.93 (3H, s)	17.8

^1^H- and ^13^C-NMR data (*δ* in ppm) were measured in CD_3_OD at 400 and 100 MHz, respectively.

**Table 3:** Inhibitory effects of isolated compounds on superoxide anion generation and elastase release by human neutrophils in response to fMLF/CB.

Compound	Superoxide Anion Generation		Elastase Release	
	IC_50_ (μM) ^a^	Inh% ^b^	IC_50_ (μM)	Inh%
**1**	– ^c^	22.4 ± 3.1 **	–	25.4 ± 6.2 *
**3**	–	8.7 ± 4.3	–	8.6 ± 1.1 **
**5**	2.3 ± 0.2	100.2 ± 1.1 ***	5.0 ± 0.3	88.2 ± 3.4 ***
**6**	–	–	–	35.1 ± 4.8 **
**8**	–	-0.7 ± 1.5	–	3.1 ± 1.6
**9**	–	-1.0 ± 2.6	–	3.7 ± 3.2
**11**	–	26.0 ± 7.8 *	–	5.3 ± 4.0
**14**	3.7 ± 0.6	86.7 ± 3.9 ***	5.2 ± 0.4	86.9 ± 6.3 ***
**15**	–	3.9 ± 4.4	–	12.4 ± 0.8 ***
**19**	4.1 ± 0.4	84.1 ± 7.0 ***	4.3 ± 0.2	94.3 ± 3.8 ***
**24**	–	13.2 ± 3.1 *	–	15.6 ± 2.3 **
LY294002 ^d^	1.1 ± 0.3	100.6 ± 1.0 ***	3.2 ± 1.0	76.7 ± 6.8 ***

Results are presented as mean ± SEM (n = 3,4). * *p* < 0.05, ** *p* < 0.01, *** *p* < 0.001 compared with the control (DMSO). ^a^ Concentration necessary for 50% inhibition (IC_50_). ^b^ Percentage of inhibition (Inh%) at 10 μM concentration. ^c^ Not determined. ^d^ A phosphatidylinositol-3-kinase inhibitor was used as a positive control.

**Table 4:** Binding energies of compounds **5**, **14**, and **19**, and LY294002 calculated in silico.

Compound	Affinity (kcal/mol)
**5**	−6.9
**14**	−7.4
**19**	−7.0
LY294002	−6.0

## Data Availability

Original data can be obtained from corresponding author upon request.

## Supplementary Materials

The following are available online. S1: Anti-inflammatory bioactivity examination; Figures S1–S18: HRMS, 1D, and 2D NMR spectra of compounds **1**–**3**.

## Sample Availability

Samples of all the isolated compounds are available from the authors.
