# Supplementary material for: Constituents from the Fruiting Bodies of Trametes cubensis and Trametes suaveolens in Vietnam and Their Anti-Inflammatory Bioactivity
Source: Molecules. 2021 Dec 2;26(23):7311. doi: 10.3390/molecules26237311 (PMC8659016; doi:10.3390/molecules26237311)

Supplementary Materials

Constituents from the Fruiting Bodies of *Trametes Cubensis* and *Trametes Suaveolens* in Vietnam and Their Anti-inflammatory Bioactivity

Yue-Chiun Li [1,†], Nguyen Thi Ngan [2,†], Kun-Ching Cheng [3,†],Tsong-Long Hwang [4,5,6], Tran Dinh Thang [2],
Nguyen Ngoc Tuan [2], Mei-Lin Yang [1], Ping-Chung Kuo [1,*], and Tian-Shung Wu [1,*]

1 School of Pharmacy, College of Medicine, National Cheng Kung University, Tainan 701, Taiwan; ycli0126@gmail.com (Y.-C.L.); l3891104@nckualumni.org.tw (M.-L.Y.)
2 Institute of Biotechnology and Food Technology, Industrial University of Ho Chi Minh City, Ho Chi Minh City 700000, Vietnam; nguyenthingan_vsh@iuh.edu.vn (N.T.N.); thangtd@iuh.edu.vn (T.D.T.); nguyenngoctuan@iuh.edu.vn (N.N.T.)
3 Taiwan Sugar Research Institute, Tainan 70176, Taiwan; a64128@taisugar.com.tw
4 Graduate Institute of Natural Products, College of Medicine, Chang Gung University, Taoyuan 33302, Taiwan; htl@mail.cgu.edu.tw (T.-L.H.)
5 Research Center for Chinese Herbal Medicine, Research Center for Food and Cosmetic Safety, Graduate Institute of Health Industry Technology, College of Human Ecology, Chang Gung University of Science and Technology, Taoyuan 33302, Taiwan
6 Department of Anesthesiology, Chang Gung Memorial Hospital, Taoyuan 33302, Taiwan
* Correspondence: z10502016@ncku.edu.tw (P.-C.K.); tswu@mail.ncku.edu.tw (T.-S.W.); Tel.: +886-6-2353535 (ext. 6806) (P.-C.K.); Tel.: +886-6-2757575 (ext. 65333) (T.-S.W.)
† These authors contributed equally to this work.

Contents

S1. Anti-inflammatory bioactivity examination.

Figure S1. HRMS & MS spectra of **1**.

Figure S2. ^{1}H NMR spectrum of **1** (CD_3OD, 400 MHz).

Figure S3. ^{13}C and DEPT NMR spectrum of **1** (CD_3OD, 100 MHz).

Figure S4. HSQC spectrum of **1** (CD_3OD, 400 MHz).

Figure S5. HMBC spectrum of **1** (CD_3OD, 400 MHz).

Figure S6. NOESY spectrum of **1** (CD_3OD, 400 MHz).

Figure S7. MS spectrum of **2**.

Figure S8. ^{1}H NMR spectrum of **2** (CD_3OD, 400 MHz).

Figure S9. ^{13}C and DEPT NMR spectrum of **2** (CD_3OD, 100 MHz).

Figure S10. HSQC spectrum of **2** (CD_3OD, 400 MHz).

Figure S11. HMBC spectrum of **2** (CD_3OD, 400 MHz).

Figure S12. NOESY spectrum of **2** (CD_3OD, 400 MHz).

Figure S13. HRMS & MS spectra of **3**.

Figure S14. ^{1}H NMR spectrum of **3** (CD_3OD, 400 MHz).

Figure S15. ^{13}C and DEPT NMR spectrum of **3** (CD_3OD, 100 MHz).

Figure S16. HSQC spectrum of **3** (CD_3OD, 400 MHz).

Figure S17. HMBC spectrum of **3** (CD_3OD, 400 MHz).

Figure S18. NOESY spectrum of **3** (CD_3OD, 400 MHz).

S1. Anti-inflammatory bioactivity examination.

1. Human neutrophil preparation

Neutrophils were isolated using a standard dextran sedimentation method prior to centrifugation on a Ficoll Hypaque gradient and hypotonic lysis of erythrocytes. Blood was drawn from healthy human donors (20 to 30 years old) by venipuncture into heparin-coated vacutainer tubes using a protocol approved by the institutional review board at Chang Gung Memorial Hospital. Blood samples were mixed gently with an equal volume of a 3 % dextran solution. Leukocyte-rich plasma was collected after sedimentation of the red cells for 30 min at room temperature. The leukocyte-rich plasma was then transferred onto 20 mL of Ficoll solution (1.077 g/mL) and centrifuged at 400 g for 40 min at 20 °C. The granulocyte/erythrocyte pellets were resuspended in ice-cold 0.2 % NaCl and lysed. After 30 sec, the same 1.6 % NaCl solution volume was added to reconstitute the isotonic condition. Purified neutrophils were pelleted and then resuspended in calcium (Ca^{2+})-free Hank's balanced salt solution (HBSS) buffer at pH 7.4 and were maintained at 4 °C before use.

2. Superoxide anion generation measurement

The assay for measuring superoxide anion generation was based on the SOD-inhibitable reduction of ferricytochrome *c*. Briefly, after supplementation with 0.5 mg/mL ferricytochrome *c* and 1 mM Ca^{2+}, neutrophils (6 x 10^5 cells/mL) were equilibrated at 37 °C for 2 min and incubated with 100 nM fMLF during preincubation with 1 μg/mL cytochalasin B (fMLF/CB) for 3 min. Changes in the 550 nm absorbance reflecting a reduction in ferricytochrome *c* were continuously monitored using a double-beam, six-cell positioner spectrophotometer with constant stirring (Hitachi U03010, Tokyo, Japan). Calculations were based on differences in the reactions with and without SOD (100 U/mL) divided by the extinction coefficient for the reduction of ferricytochrome c (ε = 21.1/mM/10 mm).

3. Elastase release assay

Degranulation of azurophilic granules was determined by measuring the release of elastase as previously described. Experiments were performed using MeO-Suc-Ala-Ala-Pro-Val-p-nitroanilide as the elastase substrate. Briefly, after supplementation with MeO-Suc-Ala-Ala-Pro-Val-p-nitroanilide (100 μM), neutrophils (6 x 10^5/mL) were equilibrated at 37 °C for 2 min and incubated with drugs or an equal volume of vehicle (0.1 % DMSO, negative control) for 5

min. Cells were activated using 100 nM fMLF and 0.5 μg/mL cytochalasin B, and changes in the 405 nm absorbance were continuously monitored to assay elastase release. The results are expressed as the percent of elastase release in the fMLF/CB-activated, drug-free control system.

4. Statistical analysis

The results are expressed as mean ± SEM. Computation of 50 % inhibitory concentrations (IC_{50}) were performed using PHARM/PCS v.4.2 software. Statistical comparisons were made between groups using Student's *t*-test. Values of $p < 0.05$ were considered to be statistically significant.

Figure S1. HRMS & MS spectra of **1**.

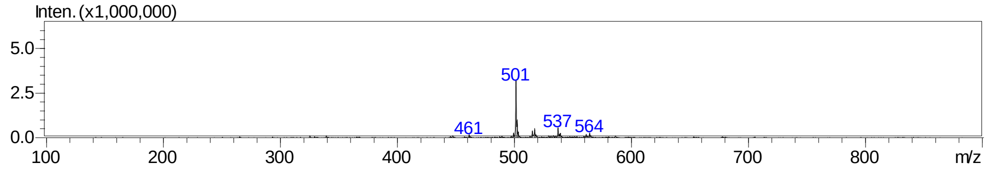

Data:Kuo_DQE_01
Comment:
Description:
Ionization Mode:ESI-
History:Average(MS[1] 0.75..0.97)

Acquired:6/9/2021 4:02:39 PM
Operator:AccuTOF
m/z Calibration File:20210520-TFANa_...
Created:6/9/2021 4:22:25 PM
Created by:AccuTOF

Charge number:1
Tolerance:200.00[ppm], 200.00 .. 200....
Unsaturation Number:-100.5 .. 200.0 (...
Element:^{12}C:30 .. 30, ^{1}H:45 .. 46, ^{23}Na:0 .. 1, ^{16}O:6 .. 6

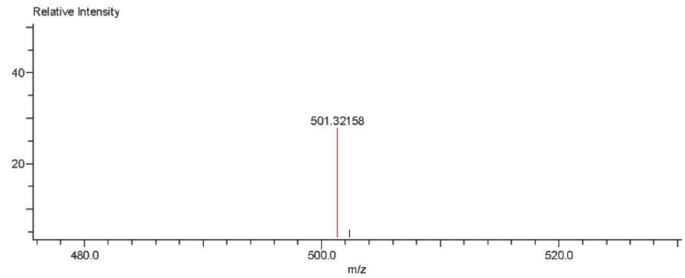

Mass	Intensity	Calc. Mass	Mass Difference [mDa]	Mass Difference [ppm]	Possible Formula
501.32158	1037.15	501.32161	-0.03	-0.06	$^{12}C_{30}{}^{1}H_{45}{}^{16}O_{6}$

Figure S2. ^{1}H NMR spectrum of **1** (CD_3OD, 400 MHz).

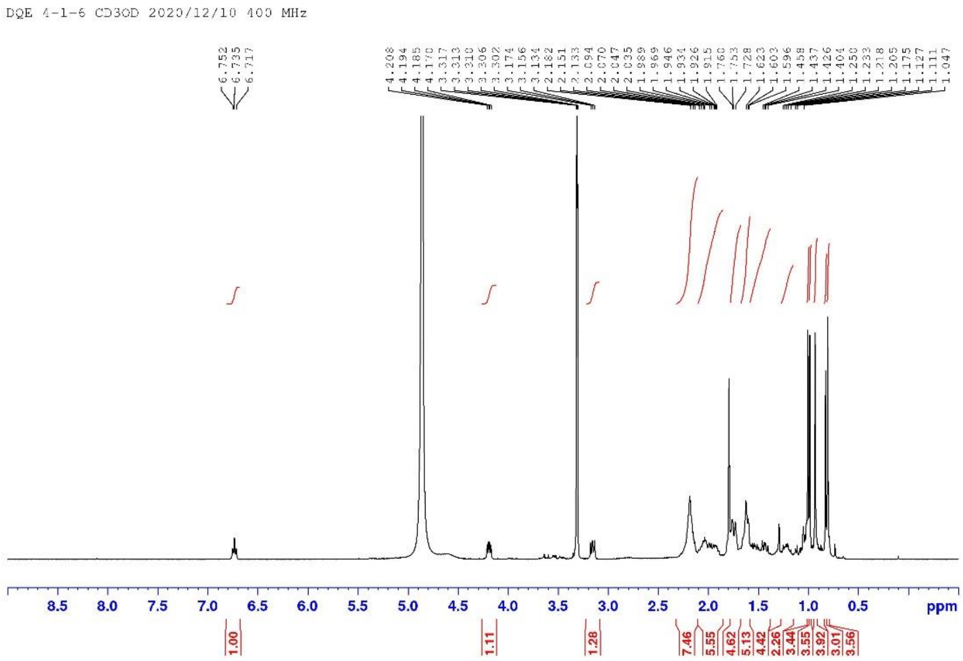

Figure S3. ^{13}C and DEPT NMR spectrum of **1** (CD_3OD, 100 MHz).

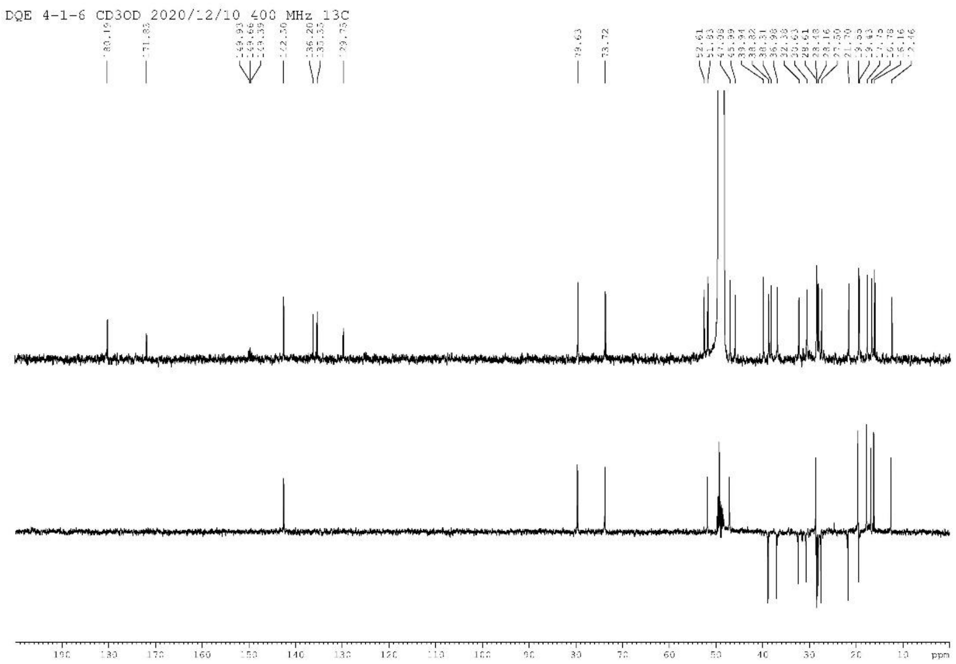

Figure S4. HSQC spectrum of **1** (CD_3OD, 400 MHz).

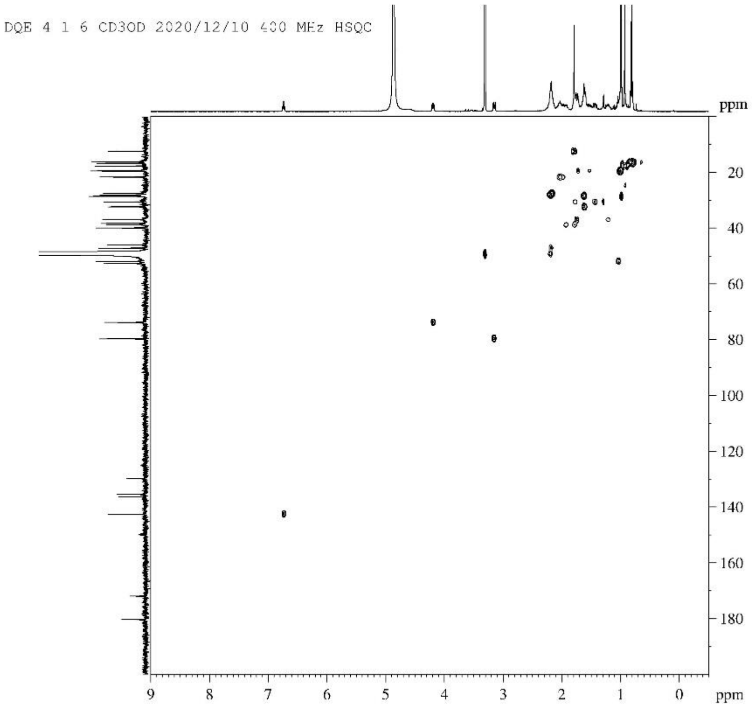

Figure S5. HMBC spectrum of **1** (CD_3OD, 400 MHz).

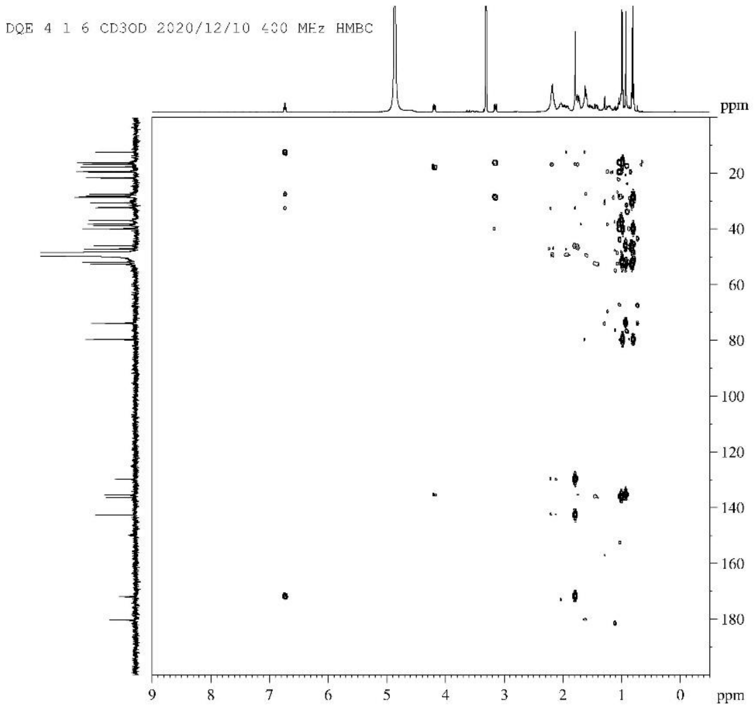

Figure S6. NOESY spectrum of **1** (CD_3OD, 400 MHz).

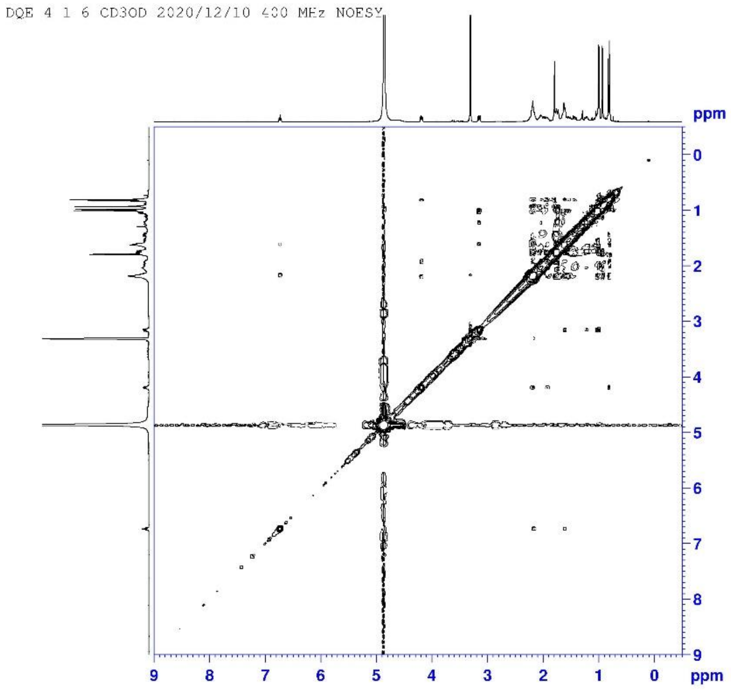

Figure S7. MS spectrum of **2**.

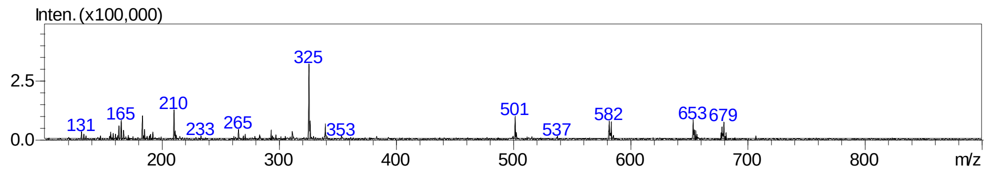

Figure S8. ^{1}H NMR spectrum of **2** (CD_3OD, 400 MHz).

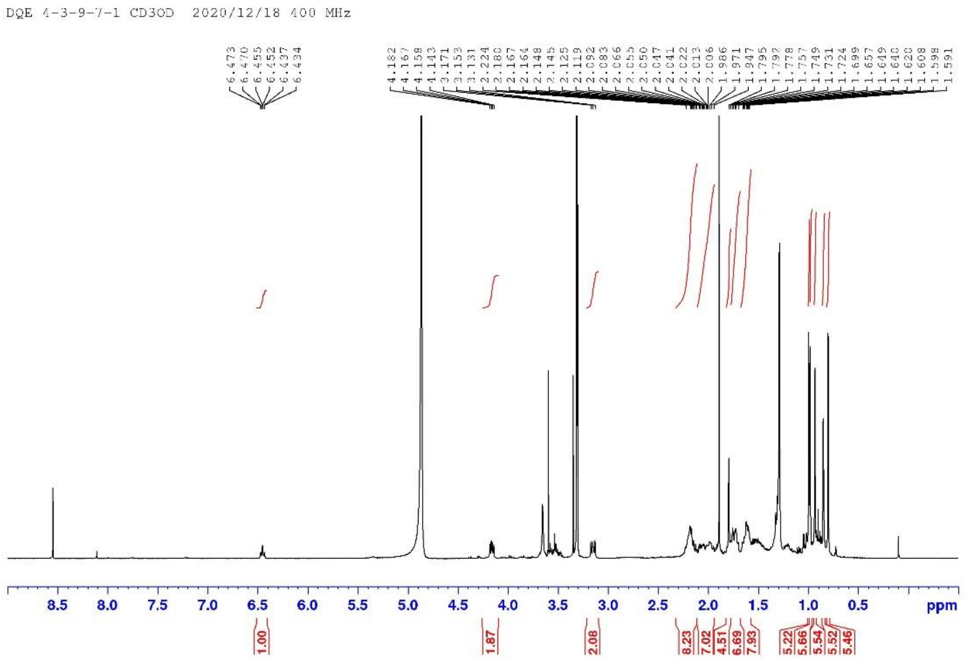

Figure S9. ^{13}C and DEPT NMR spectrum of **2** (CD_3OD, 100 MHz).

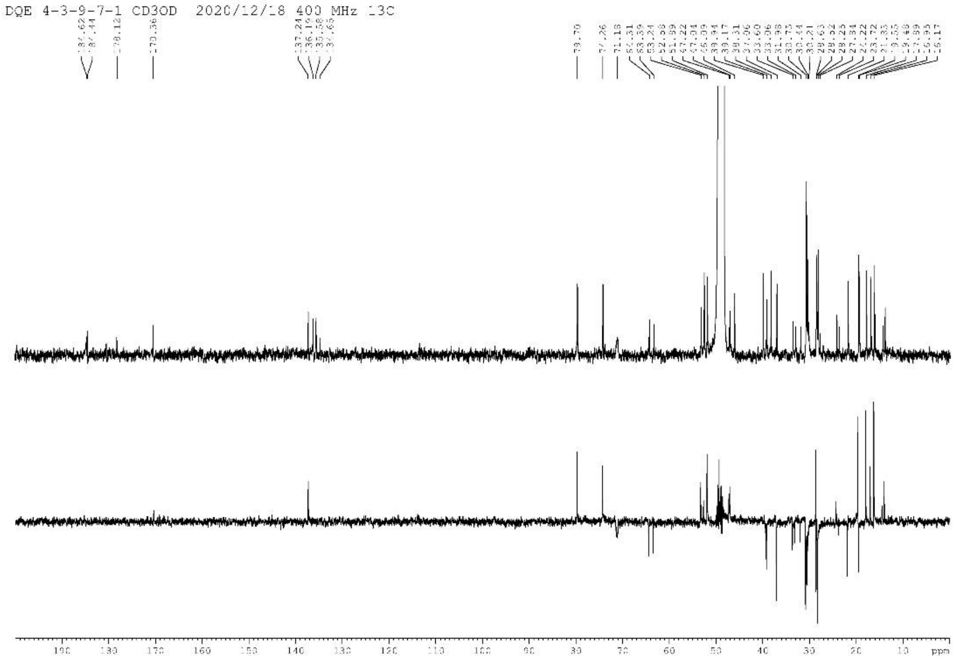

Figure S10. HSQC spectrum of **2** (CD_3OD, 400 MHz).

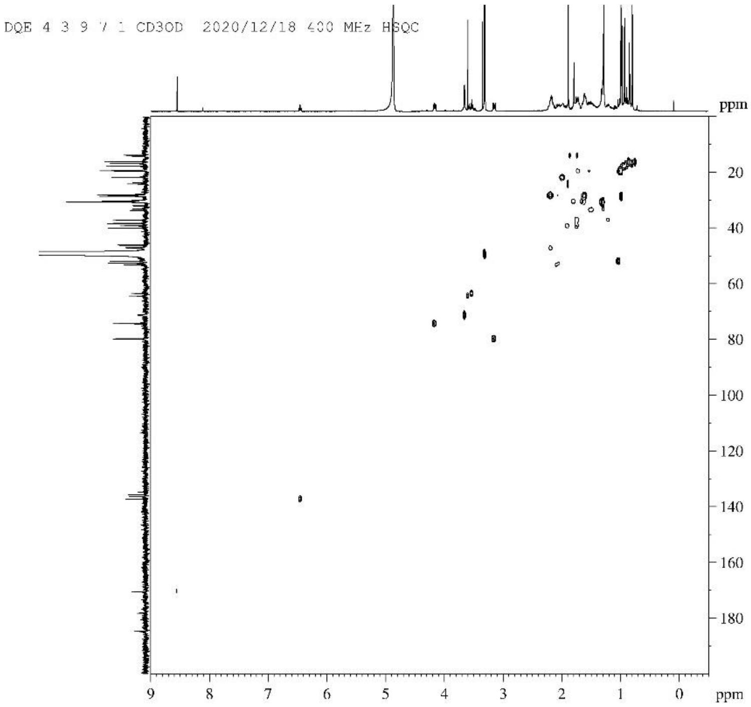

Figure S11. HMBC spectrum of **2** (CD_3OD, 400 MHz).

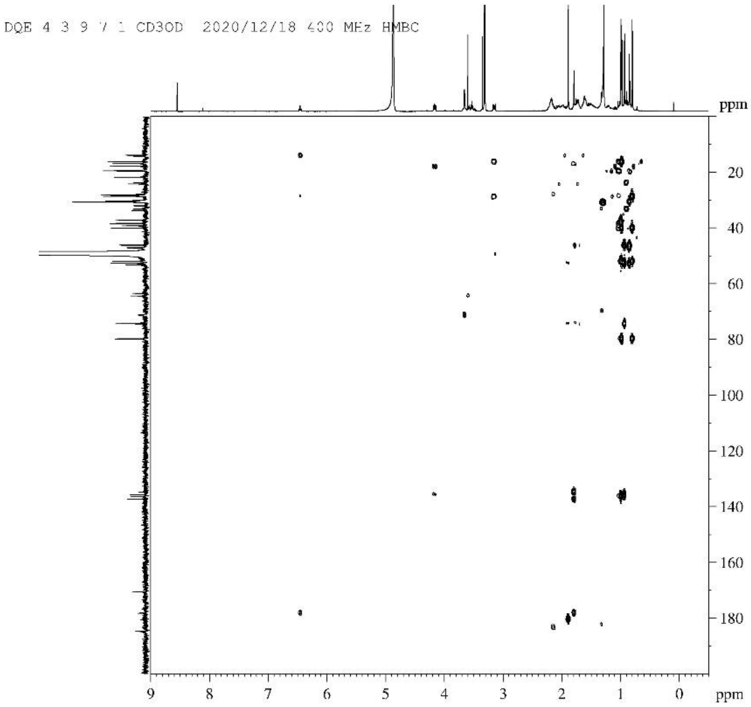

Figure S12. NOESY spectrum of **2** (CD_3OD, 400 MHz).

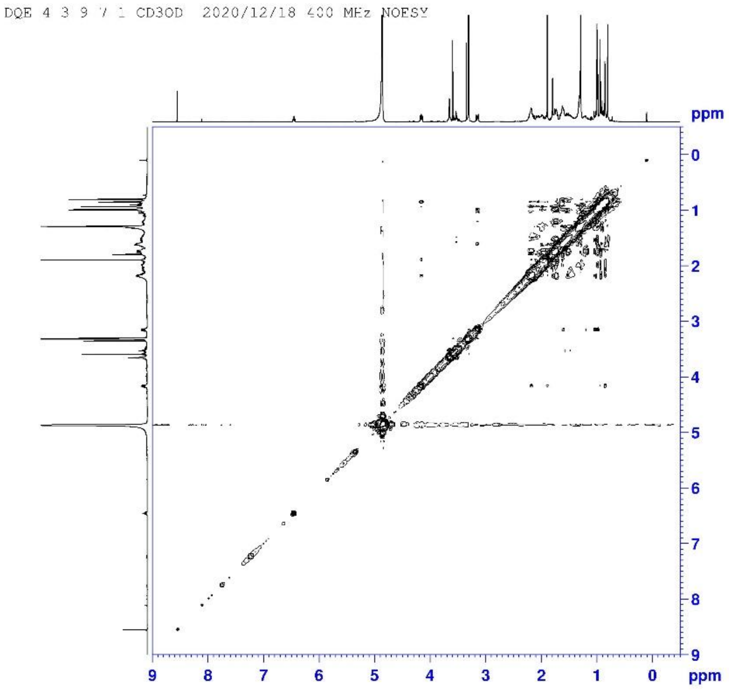

Figure S13. HRMS & MS spectra of **3**.

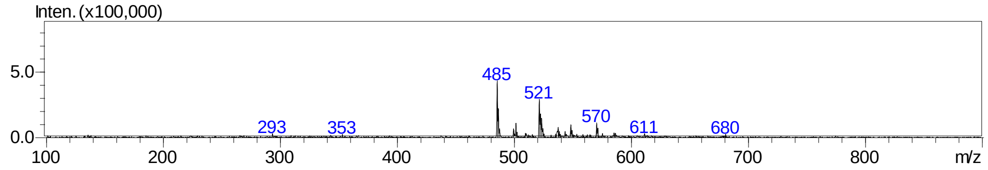

Data:Kuo_DQE_03
Comment:
Description:
Ionization Mode:ESI-
History:Average(MS[1] 0.70..0.75)

Acquired:6/9/2021 4:10:45 PM
Operator:AccuTOF
m/z Calibration File:20210520-TFANa_...
Created:6/9/2021 4:19:58 PM
Created by:AccuTOF

Charge number:1
Tolerance:200.00[ppm], 200.00 .. 200....
Unsaturation Number:-100.5 .. 200.0 (...
Element:^{12}C:30 .. 30, ^{1}H:45 .. 45, ^{23}Na:0 .. 1, ^{16}O:5 .. 5

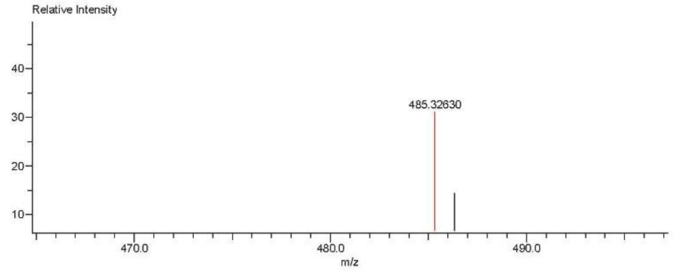

Mass	Intensity	Calc. Mass	Mass Difference [mDa]	Mass Difference [ppm]	Possible Formula
485.32630	1150.66	485.32670	-0.40	-0.82	$^{12}C_{30}{}^{1}H_{45}{}^{16}O_5$

Figure S14. ^{1}H NMR spectrum of **3** (CD_3OD, 400 MHz).

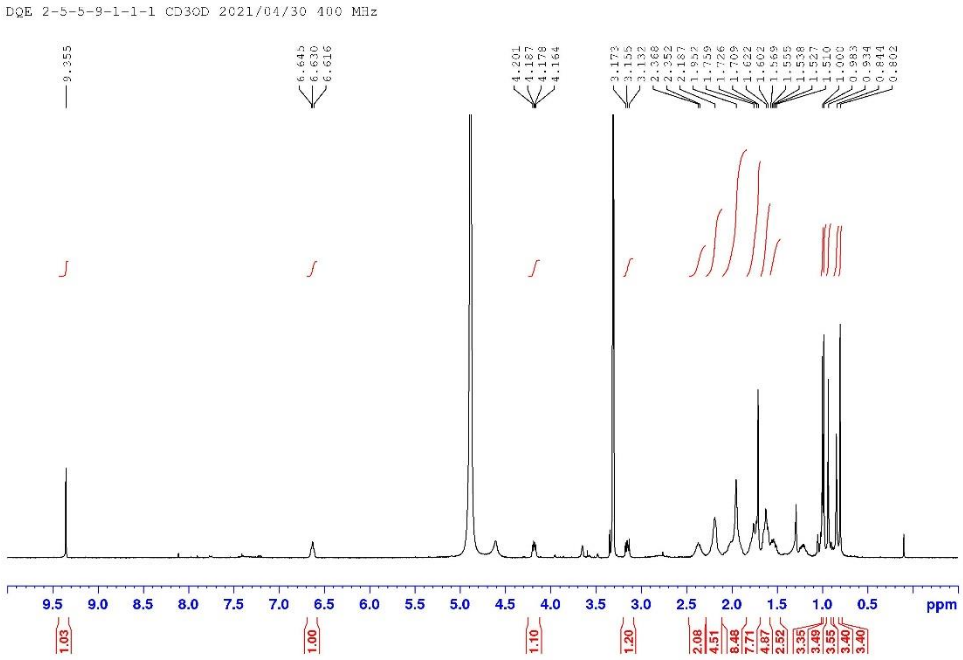

Figure S15. ^{13}C and DEPT NMR spectrum of **3** (CD_3OD, 100 MHz).

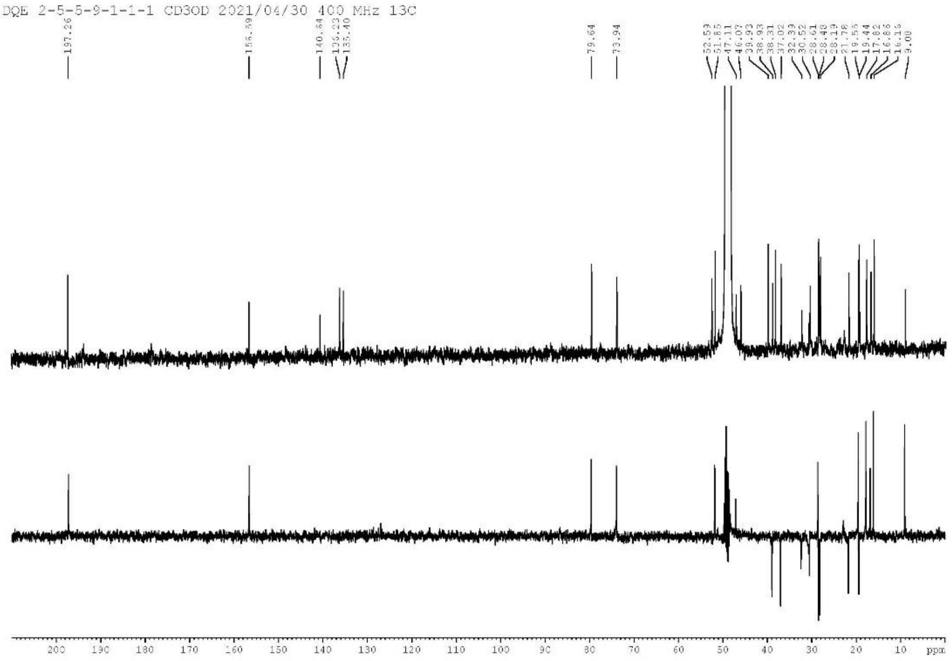

Figure S16. HSQC spectrum of **3** (CD_3OD, 400 MHz).

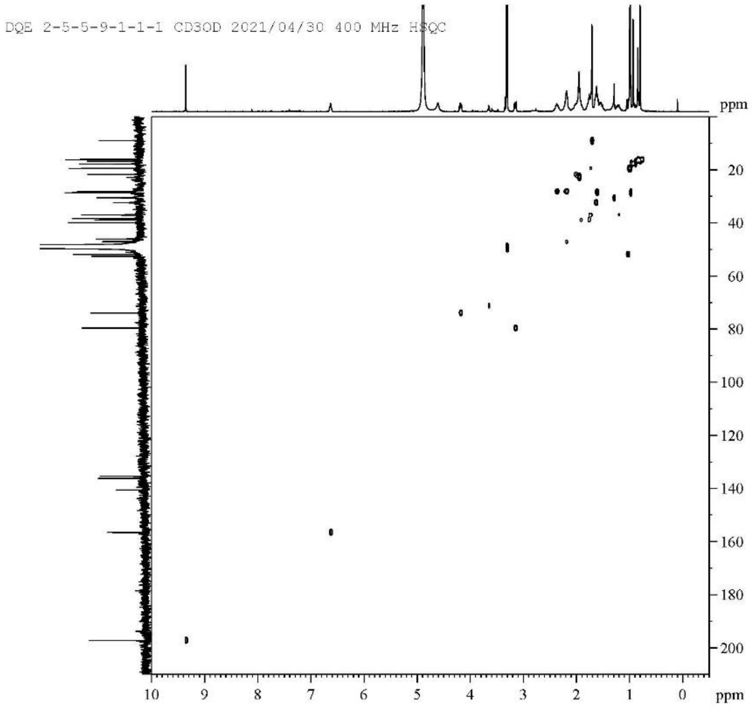

Figure S17. HMBC spectrum of **3** (CD_3OD, 400 MHz).

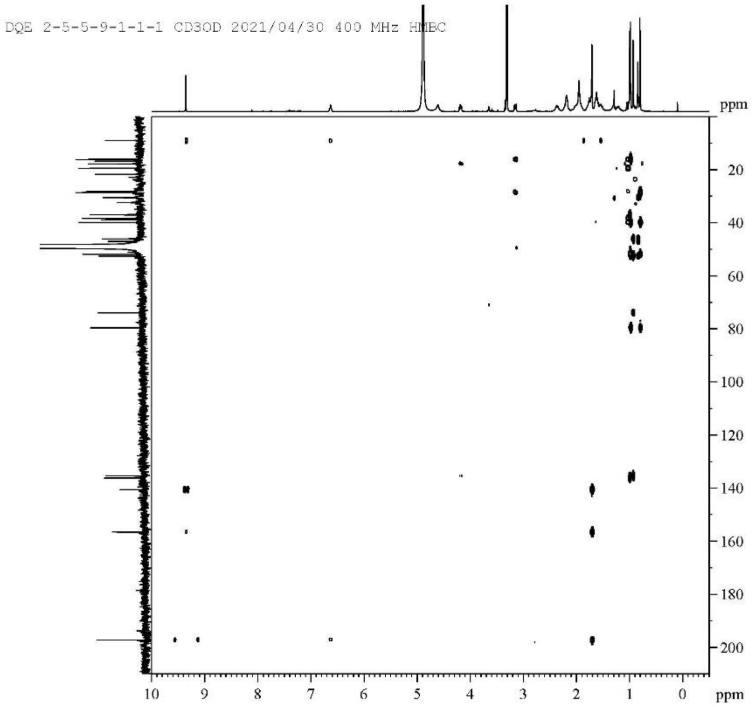

Figure S18. NOESY spectrum of **3** (CD_3OD, 400 MHz).

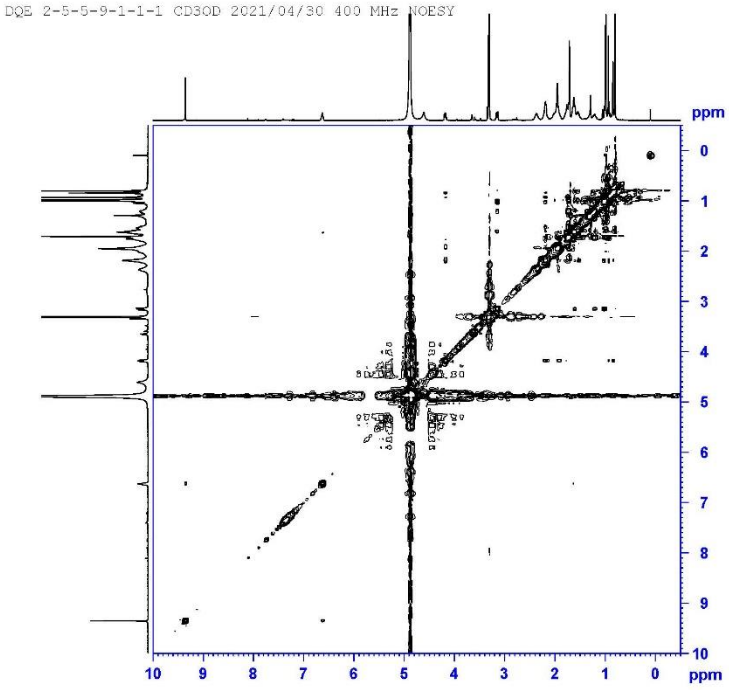

Supplement: Supplementary file 1 [file molecules-26-07311-s001.zip › molecules-1460549-SM-revised.pdf]
